# Supplementary material for: Are carbon emissions trading and green financial instruments synergistic? -Comprehensive quantitative research based on content analysis
Source: PLoS One. 2024 Mar 7;19(3):e0298601. doi: 10.1371/journal.pone.0298601 (PMC10919601; doi:10.1371/journal.pone.0298601)
Supplement: S1 Data — (ZIP) [file pone.0298601.s001.zip › Data/地级市Z_F工作报告.zip!/║┌┴·╜¡/╝╤─╛╦╣╩╨/└┤╫╘╣1⁄2╓┌║┼í╛┬φ┐╦╩2╛▌═°í┐_2015.docx]

各位代表：

　　现在，我代表市人民政府向大会报告工作，请予审议，并请各位政协委员提出意见。

　　一、2014年工作回顾

　　过去的一年，受国内宏观经济“三期叠加”和省内经济下行的影响，我市经济出现近年来少有的不利因素。在省委、省政府和市委的坚强领导下，我们紧紧把握当期发展的阶段性、规律性的趋势特点，围绕建设富裕文明和谐幸福佳木斯的目标，深入实施“五大规划”发展战略，通过全面深化改革、扩大有效投资、促进产业升级等措施，主动适应新常态，着力提升政府服务效能，全市经济平稳发展，民生持续改善，主要指标增速位居全省前列，经济发展的基础地位更加巩固，总体质量趋向改善。全年实现地区生产总值792.5亿元，增长6.8%，位居全省第四位；公共财政预算收入42亿元，增长0.4%，位居全省第六位；固定资产投资483亿元，增长13.5%，位居全省第二位；社会消费品零售总额361亿元，增长13.5%，位居全省第二位；城镇居民人均可支配收入21518元，增长10.7%；农村居民人均可支配收入12326元，增长11.2%。单位GDP能耗下降3.52%。

　　——产业项目建设步伐坚实。实施新一轮产业项目三年攻坚战，全市开复工千万元以上产业项目586项，完成投资343.6亿元，占固定资产投资的71.1%，项目竣工370项。装备制造、农副产品加工、现代服务业和对俄贸易加工四大类优势产业亿元以上项目占比80.5%，完成投资169.1亿元，项目质量不断提高。在对我市重点领域投资潜力深入研究基础上，谋划306个前期论证充分、资源优势突出、政策契合度较高、市场前景看好的入库项目，项目建设的系统性、实效性进一步增强。实行专业招商与全员招商相结合，引进亿元以上项目120个，域外引资到位额432亿元，增长20%，直接利用外资2.3亿美元，增长10%。佳木斯毅德城、佳大胡商商业广场等重大投资项目签约落地。全市重点建设的10个产业园区新增用地73公顷，基础设施新投入9.1亿元；新开工建设千万元以上项目42个，完成投资70亿元，新增投产企业20户。

　　——农业现代化水平不断提升。两大平原综合配套改革逐步深化。创新农业经营主体，土地流转面积、规模经营面积分别增加130万亩、100万亩；创新农村金融，引导县乡村三级土地流转服务平台与农村金融服务有机结合，推出新型金融产品14项，发放涉农信贷新品种贷款24亿元，贷款利率稳中有降；全市仓储设施增容400万吨，新增烘干能力180万吨，为粮食加工转化奠定了基础；引进厦门象屿等21家农业企业，规模以上龙头企业引领带动29万农户参与产业化经营，销售收入增长20.5%；组建全省首家优质农产品行业协会，“佳木斯大米”地理标识通过农业部认定；在沈阳、大连建立绿色特色农产品销售专区和社区网点，引导农产品销售增值模式实现新突破。绿色特色农业加快发展。国家级绿色食品标准化生产基地面积达到830万亩，同江市在全省率先建立绿色食品质量安全管理追溯系统；食用菌栽培规模达到7.1亿袋，增长18.3%，形成市场+龙头+基地+农户的产业链模式；畜牧业、林业、渔业产值分别增长10.8%、8%、21.2%。农业基础设施建设得到加强。推进智能化催芽基地、水稻集中育秧小区、标准化育秧大棚建设，保证80%以上水田的优质秧苗供应；投入水利建设资金10.4亿元，在增强应急度汛能力基础上，新增灌溉能力27万亩。全市农业科技贡献率超过63%，综合农机化程度提高到94.2%，粮食总产140.3亿斤，实现“十一连增”，商品率达到90%。

　　——工业经济保持稳中有进。推进企业梯次成长升级，新增规模企业16户、小微企业18382户。开复工投资5000万元以上重点工业项目123项，完成投资208.6亿元，增长18.6%，名列全省第一。百威英博(佳木斯)公司年产100万千升啤酒一期、佳木斯中唯实业公司模具冲压中心等项目开工建设，郑龙煤机液压支架和刮板机、三和亚麻公司5万锭亚麻纱等项目投产运行。大力支持企业开展技术创新、产品创新，电机股份有限公司的核电K1类电机等一批新产品技术水平全国领先，东北林大的轻量型节能保温阻燃环保材料等一批技术成果在我市落地转化，高新技术产业增加值占GDP比重为7.8%。县域经济规模不断壮大，产值占全市经济总量的69.1%。全市实现规模以上工业增加值94.6亿元，增长4.7%，居全省第四位。

　　——服务业发展势头良好。完善商业功能，佳木斯万达城市广场项目开工建设，桦南招远皮革城、大润发英伦万新店等项目投入运营，京东商城、58同城等大型网络企业分支机构落户我市，引领拉动城乡和周边地区消费升级。突出特色旅游资源，打造精品组合线路，全市接待境内游客391万人次，旅游收入16.1亿元，增长18%。促进服务外包业务开展，新增商务部备案的服务外包企业2户，产值增加15%。银河证券等金融机构进驻我市，在全省组建首家农村金融服务公司，金融机构存贷比达76.96%，金融产业实现税收4亿元，增长9.1%。

　　——合作开放活力持续增强。中俄同江铁路大桥、哈佳快速铁路等一批重大基础设施项目开工建设。佳木斯至首尔航班转为正式航班，佳木斯至哈巴航线加班飞行，佳木斯至深圳航线实现通航，全年空港旅客吞吐量突破47万人次，增长12.7%。加强境外合作项目建设，在俄投资增长12.5%。首次实现俄罗斯石油液化气进口，佳天国际农副产品物流交易中心海关监管库获批设立。与俄罗斯地方政府间多次组团互访，并为企业家、艺术家搭建交流平台，互派留学生115人，密切了双方经贸合作与人文交流。成功举办2014第五届中俄(佳木斯)农机展销洽谈会，增设产业论坛、产品推介等活动内容，展会与产业融合度明显提高。全市进出口总额完成25.4亿美元，增长12%。

　　——城乡建设统筹推进。坚持规划引领，制定实施专项规划、城市景观风貌规划等238项。新建续建道路15条，解放路立交桥、胜利西路至鹤大高速正式通车；完成42条主要街道及61条巷道维修改造、52条区街巷道建设任务，道路完好率达到98%以上，道路维护实现常态化，城市交通、百姓出行更加便利。深入开展9项专项整治活动，城区“脏乱差”和占道经营等问题得到改善。市区新增供热面积160万平方米，并网小锅炉57台，完成天然气安装1.7万户。投资16.7亿元，完成城镇化和中心村试点年度建设任务。灾后水毁重建工程顺利完工。郊区探索的美丽乡村建设经验，得到省委、省政府充分肯定。推进松花江水污染防治项目，松花江干流水质达标率100%。在全省率先实现所辖县(市)全部晋升为国家级生态示范区。全市植树造林1.1万公顷，村屯提前两年全面完成省下达的护村林建设任务。主要污染物减排连续三年全省第一。

　　——惠民利民工作成效显著。全市民生投入115.8亿元，占公共预算支出的61.7%，年初确定的20件民生实事全部完成年度目标。城镇新增就业7.4万人。养老、医疗、失业保险扩面超额完成年度任务；新农保与城居保账户实现合并，新农合筹资标准提高到390元。城市低保标准提高到440元/月，农村低保标准提高到2700元/年。农村五保集中供养率50%。全市新开工保障房1595套、11.59万平方米，分配廉租房300户，累计发放廉租补贴4209万元。改造农村泥草房1.2万户。解决农村8.6万人饮水安全问题，推进4.3万农村贫困人口脱贫。年增513万元提高环卫工人工资标准。改善中小学办学条件，维修校舍23.6万平方米，义务教育阶段实现阳光招生、阳光分班。深入开展医疗合作“五联工程”，40项新技术、新项目填补省市空白。各级公共图书馆、博物馆免费开放。群众体育工作进入全国先进单位行列，我市被省体育局授予全省唯一的“全省全民健身城”。完成城区社区综合体制改革，启动74个规范化城市社区三年达标工程。安全生产形势和社会治安总体稳定，群众满意度持续提高。食品药品安全监管形势稳定向好，在2014年国家组织的四大类农产品质量安全检测中，是全省唯一合格率为100%的城市，连续三年被评为中国十佳食品安全城市。投入资金4500余万元化解信访积案，长期积累的一些社会矛盾得到有效缓解。

　　——政府自身建设得到加强。切实改进作风。深入开展党的群众路线教育实践活动，确定30项整改问题、16项专项整治内容，在23个方面建章立制，巩固扩大了活动成果。加快职能转变。累计下放行政职权587项，行政职权精减率达到46.4%，在全国市(地)级城市中率先公布权力清单；市政务服务中心投入使用，本级审批流程全部优化，审批提速60%以上；清理收费项目30个，减少收费2100余万元，政府工作提速提效、便民惠民。坚持依法行政。建立市长例会等制度，健全完善规范性文件、对外合同协议、行政决策合法性审查机制，重大事项集体讨论，集思广益；成立土委会、规委会等议事机构，理顺议事议程，扎紧制度笼子。健全政务公开、定期新闻发布制度，自觉接受监督，人大代表建议、政协委员提案答复率100%，满意率分别为90%、99%。严格公共资源管理，全市节约采购资金5134万元，清理超标、闲置办公用房3.1万平方米，清理吃空饷727人，市直“三公”经费下降20.4%。加强反腐倡廉工作，查处各类违纪违法案件507件，有效净化了行政服务环境。

　　一年来，行政监督、市场监管、外事侨务、民族宗教、机关事务、统计、气象、地震、人防等工作得到加强，妇女儿童、残联、红十字等工作扎实推进，中省直部门、驻佳部队为地方发展做出了积极贡献。

　　各位代表，过去一年，我们的成绩来之不易。这是在市委正确领导，市人大、市政协鼎力支持下取得的，是全市上下广大干部群众团结一心、攻坚克难、奋力拼搏的结果。在此，我代表市人民政府，向各位代表和全市人民，向离退休老领导、老同志，向各民主党派、工商联、人民团体，向各位企业家、科技工作者，向驻佳部队和武警官兵，向各中省直单位，向所有关心、支持佳木斯发展的各界人士、国际友人和海内外佳木斯籍人，表示崇高的敬意和衷心的感谢！

　　回顾一年来的工作，经济社会发展中还存在着不容忽视的问题。主要表现在：经济总量不大，第一产业与二、三次产业融合度不高，初级产品多、附加价值少，二产比重偏小的长期性问题依然存在；产业建设招商的针对性不强，投资强度低，部分项目投产达效慢，投资环境问题仍然较多；群众关心的上学、看病、就业、供暖等民生事业总体投入大，但问题解决得不够平衡，服务还不到位；政府部门用市场机制配置公共资源的理念、能力还不强，公共资源的损失浪费不容忽视；部分公职人员工作责任心、专业化水平、工作作风等与新常态要求和人民期盼还有差距。这些问题，我们将牢记于心，努力破解。

　　二、2015年主要工作

　　2015年，我市经济发展的趋势性变化会更加明显，需要我们以辩证的思维分析形势，把握机遇。从不利因素看：粮食加工企业、“两头在外”工业企业、行业产能过剩企业等市场主体，面临市场和政策因素的持续影响，经营压力会更大；房地产市场信心不足、普通商品房滞销，房地产行业资金周转、稳定经营的形势会更加严峻；政府性融资政策收紧，政府性陈欠资金的偿还渠道将受到严格限制，财政现金流面临困难。从有利因素看：在新常态下，我市绿色生态资源、优质商品粮资源，现代农业产业化发展趋势和我市农业规模化、组织化程度较低的潜力，将会受到更多潜在投资者的关注；重大基础设施建设、对俄合作区位优越、生产要素集聚等比较优势将会给承接产业转移带来更多机会；多年来积蓄的发展基础和新建产业项目产能规模效应的显现，将形成多点支撑拉动能力；去年以来，国家和省政府出台的各项政策和重点规划，将为我们提供谋划更多项目、向上争取更大资金的机会；特别是随着改革的全面深化，必将在政府管理、产业发展、投资环境等方面激发和释放更大活力。我们必须始终以积极进取的姿态和奋发有为的精神，抢抓新机遇，迎接新挑战，着力破解发展中遇到的困难和矛盾，推动全市经济社会持续健康发展。

　　今年政府工作的总体要求是：全面贯彻落实中央和省委、省政府的工作要求，按照市委的部署，主动适应经济发展新常态，坚持稳中求进工作总基调，以提高经济发展的质量和效益为中心，发挥自身优势，注重创新驱动，在扩大有效增量中优化结构，促进三次产业融合发展；以问题和需求为导向，狠抓改革攻坚，深化区域合作，积极改善民生，推进依法行政，促进经济平稳健康发展，社会和谐稳定。

　　综合考虑宏观经济发展的规律性趋势，以及我市经济的自身特点，本着积极进取的态度，今年主要经济指标预期是：地区生产总值增长6.5%左右，固定资产投资增长10%左右，社会消费品零售总额增长13%左右，城镇、农村常住居民可支配收入分别增长9%、10%以上。

　　(一)突出质量效益，务求产业项目建设实效。把握项目建设谋划、招商、推进三个阶段性规律，加快建设引领产业方向、拉动税源增长、支撑经济快速发展的大项目、好项目。新谋划储备产业项目300项，开复工500项，其中新开工300项，竣工投产200项，实现产业项目投资稳中有升。完善项目建设布局统筹和推进落实机制，注重提高投资强度和产出效果。力争百威英博(佳木斯)公司年产100万千升啤酒一期、泉林生态农业公司秸秆综合利用一期等重点推进项目建成投产，佳木斯毅德城、通用航空基地等重大投资项目开工建设。突出京、津、沪、鲁等重点区域，充分借助各类有效载体，立足优势潜力，创新招商方式，开辟引资本、引技术、引人才有机结合的新路径，国内招商引资到位资金、实际利用外资均增长10%以上。加快园区基础设施建设，统筹要素资源，调整利益机制，强化综合服务，引导产业集群集聚，推动市高新区和富锦绿色食品产业、同江进出口加工等县(市)园区特色发展、集约发展、持续发展。市高新区在完善配套功能规划、提升综合承载能力的基础上，重点培育以现代农业为依托的泉林循环经济产业，谋划建设综合物流园区，争取开发区升级为国家级园区；全年完成产业项目投资35亿元，建成投产项目15项，销售收入增长15%以上。

　　(二)转变发展方式，加快农业转型升级步伐。以深化两大平原综合配套改革为牵动，推动我市现代农业资源优势加快向产业和经济优势转变，促进粮食稳产、农民增收、产业创税。全面落实“五个创新”和新“四化”的要求。创新农业经营主体，培育合作经济组织200个，发展多种形式适度规模经营；建立产权流转交易市场，完成农村土地确权登记，规范土地流转行为，探索农村集体经营性建设用地入市；支持各类合作经济组织与龙头企业加强资本、科技、品牌、管理等要素联结，增强持续盈利和抗风险能力，全市规模以上龙头企业产值增长15%以上；构建社会化服务体系，提高种业研发、农资供应、金融保险等方面的服务水平，加速区域内农机、农技、水利的深度融合，实现粮食增产3%以上。拓宽农产品流通渠道。加快象屿集团粮食仓储物流、北大仓佳木斯国际粮食物流交易中心建设，形成辐射三江地区、连接国内外销售市场的优质、安全、绿色农产品交易网络，引导农产品贸易主体属地化注册经营，培养税源增长点；大力发展电商与实体流通相结合的现代农产品物流体系，启动“佳木斯大米”地理标志品牌的营销工作，争取进入以全国主流超市为核心的营销网络，真正从“种得好”向“卖得好”转变。瞄准市场需求，推动特色农业规模化、产业化、品牌化发展。强化食用菌技术指导和市场服务，扩大育秧大棚综合利用，黑木耳、香菇等食用菌栽培规模达到7.6亿袋；杂粮、果蔬、紫苏等特色种植面积达到40万亩以上，完成“桦南紫苏”原产地保护产品使用地理标识申报；名特鱼养殖基地发展到50个，新建及改扩建“两牛一猪”标准化规模养殖场50个。建立农产品种植生产标准指导体系，引导和规范农药、化肥等农业投入品施用行为，完善农产品质量监管追溯体系，把“优质、安全、绿色”作为我市农业产出品的重要战略品牌。加强农业生态保护，从战略高度重视保护森林、保护湿地、保护黑土，发展可持续农业。培养新型职业农民和农村致富带头人，拓宽农民增收致富的新渠道。推动场县合作共建由种植领域向农产品精深加工、园区合作、品牌培育等多领域延伸，实现优势互补。推进防洪减灾，灌区续建配套和田间排灌、节水改造项目，探索农田水利设施市场化运营模式。

　　(三)引进有效增量，提高工业经济竞争实力。坚持大中小企业一起抓，在做大做优总量中优化结构，规模以上工业增加值增长4.5%左右。向优势存量要增量。制订差异化的激励政策，重点支持电机公司、沃尔德电缆等骨干企业、市场占有率高的行业龙头企业裂变升级，做大做强。鼓励有条件的中小企业参与行业重组、合资合作、上市融资，有效破解市场、资金、技术等发展瓶颈问题，新增规模以上企业12户。发挥生产要素协调机制作用，促进常发佳联等有潜力的停产、半停产企业恢复生产，帮助东兴煤化工、佳星玻璃等困难企业度过难关。向一产延伸要增量。发挥绿色优质农副产品量大质优、商品率高的优势，加快发展食品及农副产品精深加工，建设食品工业产业集聚区。发挥农机产业的基础竞争优势，引导农机企业顺应市场需求变化，调整产品结构，提高产品性能，扩大市场份额，激发农机产业新活力。发挥中心城市辐射范围广、消费拉动强、区域农资消费未来预期潜力大的优势，发展肥料、饲料、新型农膜等农业投入品的专业化生产，培育农资产业的新增量。发挥区域秸秆资源丰富的优势，创新综合利用技术路径，重点推动佳木斯泉林秸秆综合利用项目的产业化扩张，打造现代农业循环经济的新亮点。向大众创业要增量。全面落实支持民营经济和中小微企业发展的政策措施，发挥中小企业服务平台和各类产业孵化器的作用，放开准入领域，新增小微企业1.3万户以上，民营经济增加值增长7%以上。实施“人才强企”战略，培养高素质的企业家队伍，营造尊重、保护优秀企业家的良好环境。向科技创新要增量。加强知识产权保护与转让市场体系建设，借助省科技服务平台，推动大专院校、科研单位技术与企业资本的深入对接，促进科技成果资本化、产业化。重点引导扶持小微企业向“专、精、特、新”方向发展，最大限度释放创新活力。高新技术产业增加值占GDP比重达到8.5%以上。

　　(四)优化业态结构，促进服务业提档升级。充分发挥区域中心城市优势，进一步完善现代服务业体系，促进服务业发展提高速度、提高比重、提高水平。升级发展消费性服务业。加快推进万达国际广场、利达国际购物中心等大型商贸项目，同步引导发展农村服务业。培育发展一批具有现代交易模式的网络加盟店和连锁店，积极拓展集时尚娱乐、休闲文化、便利服务于一体的，具有城市主题特色的综合性高端消费，不断丰富新兴商贸业态。大力发展生产性服务业。重点提高生产资料市场、现代物流、研发设计、节能环保服务、电子商务、服务外包、融资租赁、人力资源服务等领域对相关产业的综合服务能力。加快发展文化旅游业。按照整合资源、点上突破、线上提升的思路，加大城市旅游资源形象刻画和宣传力度，加强汤原亮子河，桦南七星峰，富锦湿地，同江三江口、赫哲村寨，桦川星火民俗风情园，抚远黑瞎子岛，郊区猴石山、敖其湾等重点景区建设，晋升4A级、3A级景区各1家。筹建日军侵华物证陈列馆、佳木斯知青纪念馆、中俄油画馆，推进演艺公司以市场为导向的常态化演出，鼓励文化创作，凝练文化成果，繁荣文化市场，促进地域文化与特色旅游资源融合发展。规范文化旅游市场管理，完善旅游服务机制，全年文化旅游产业总收入增长15%以上。鼓励发展养老服务业。突出公益性基本养老和老年健康养生服务，完善社区养老功能，利用闲置培训中心等非办公类资产，建设3个市场化健康养老服务项目，引导养老服务业向事业型和产业化并重转变。支持发展金融产业。推动广发银行、中信银行、兴业银行进驻运营，完成佳木斯农村商业银行筹建，支持银行资本、社会资金组建村镇银行，发展私募股权投资基金和风险投资基金，以有效竞争满足融资需求，降低融资成本。规范民间借贷，打击偷逃债务行为，净化金融环境。全市金融业实现税收4.42亿元，增长10%以上。优化调整房地产业。推动江山组团等4个棚户区改造项目，强化棚改资金管理，建立以商品房开发用地为重点的土地收储与棚改融资闭合运行的管理模式。依法推进逾期回迁房地产项目加快建设，积极引导存量商品房加快销售，谋划商业、旅游文化地产项目，优化房地产结构。

　　(五)深化专项改革，激活发展内生动力。按照市委部署，坚持问题导向，推动重点领域和关键环节改革取得新成效。深化公共资源市场化配置改革。建成公共资源交易平台，规范交易程序和交易制度，真正使市场在政府工程、土地出让、特许经营权转让等公共资源交易和配置的过程中起决定性作用，防止政府权力寻租。推广政府购买服务，让市场主体更多更好地分享到改革创造的商机。深化行政审批制度改革。加强对事业单位、相关中介机构等实质性行政权力的清理规范，公示权力清单，推行责任清单，解决好审批事项“接管放”中权责界定问题。动态调整和精简审批事项，推行网上审批，完善投诉机制，加强事中事后监管，最大限度提高审批效率。深化财政预算绩效管理改革。努力构建全口径预算管理体系，形成严格规范、公开透明的预决算管理制度；将绩效理念融入预算管理全过程，把增收节支效果作为绩效评价的重要指标，强化绩效跟踪和评价结果应用；建立科学合理的政府债务管理和风险预警机制；优化行政事业经费支出结构，全面清理财政专户资金，严格控制“三公”经费。深化事业单位、国有企业改革。加强国有资产、国有资本经营管理，推动事业单位市场化改革；盘活利用闲置国有资产向经营创效领域转变，开展国有企业、国有资产经营管理绩效审计，防止国有资产流失。积极稳妥推进机关事业单位养老保险制度改革和厂办大集体改革试点工作。继续深化文化体制改革。

　　(六)抢抓战略机遇，实现对外开放新突破。以贯彻国家“一带一路”战略规划、我省加快建设“中蒙俄经济走廊”黑龙江陆海丝绸之路经济带为契机，全力打造佳富同抚对俄沿江开放试验区，形成多层次、多领域、跨境互动的对俄开放合作新格局。积极推进中俄同江铁路大桥、哈佳快速铁路建设，适时启动5条铁路项目前期；开工建设4条国、省道干线公路，加快推动“三江连通”工程。谋划建设5个对俄合作境内外园区，支持优势企业“走出去”，在俄投资增长13%以上。抓住俄罗斯实施远东大开发的机遇，支持本地企业积极参与俄方基础设施建设和农业开发。务实办好中俄(佳木斯)农机境外展。构建对俄多元化的人文交流体系，在增进相互尊重认同中促进更多领域合作。落实口岸通关便利化措施，提高地方产品出口比重，全市外贸进出口总额增长3%左右。

　　(七)注重统筹协调，打造宜居城乡环境。按照“一次性规划、全区域覆盖、多层次使用、立体式管理，模块化建设”的要求，启动新一轮城市总体规划修编，实现城市总体规划、专项规划、详细规划有序衔接。以完善城区路网、方便群众出行为重点，推进15条城市主干路、3条高新区道路的续建新建，45条主次干道、50条区街巷道的维修改造工程。开工建设佳西客运枢纽站。城区新建公厕22座，新增停车泊位2000个；调整延伸公交线路5条，满足市民出行需求。整合现有数据资源和专业管理需求，加快智慧城市统筹建设步伐。深入开展城区环境卫生、牌匾亮化、扰民污染等专项整治，实现城市面貌和品位的改善提升。抓好松花江污染防治，推进英格吐河水体污染综合整治等7项工程。完善城市内河防洪规划，实施高新区防洪工程建设。加快127个城中村城镇化改造和7个试点镇、20个试点村的城镇化进程，铺装村镇街路145公里，建设农村公路213公里。抓好饮用水源地保护，完成城乡绿化造林面积8400公顷。实施村庄建设“三项工程”，切实改善农村人居环境。

　　(八)坚持需求导向，持续推进民生改善。立足为基层群众解决实际问题，让群众在保基本、救急难、便利化的服务中真切感受到党和政府的为民情怀。提高保障水平。城镇医疗、工伤、生育保险继续扩面。调整全市城镇职工基本医疗保险缴费比例和支付标准。提高失业金发放和城乡低保、五保供养标准。新增养老床位2800张，农村五保集中供养能力达到70%以上。突出“救急难”，健全和完善临时救助制度。完善城乡均等的公共就业创业服务体系，城镇新增就业5.6万人。推动各类企业规范合法用工，建立农民工工资行业负责制，严厉打击拖欠农民工工资行为。加强农村劳动力专业化职业培训，实现劳动力转移35.75万人。全市改造棚户区19636户、152.6万平方米，建设公租房700套，改造泥草(危)房6000户。解决113个村屯7.55万人口的饮水安全问题。完成农村2.5万贫困人口脱贫目标。城区改造老旧小区楼房650栋，动态实现弃管楼管理全覆盖；改造老旧供热管网20公里，并网小锅炉32台，49万平方米；安装天然气居民用户1万户。公积金归集、使用稳定在双10亿以上。完善公共服务。规范办学行为，促进教育公平，着力解决大校额、大班额，提高农村师资水平，公办在职教师有偿办班补课等问题；启动实施第二轮学前教育三年行动计划，新建改扩建公办幼儿园16所，农村学前教育普及率达到74%；推进教育信息化建设和应用，扩大优质教育资源覆盖面；以市场需求为导向，提高职业教育特色化办学水平。加强与国内外名院名医合作交流，提高医疗救治能力；开展“健康龙江行动”，倡导健康生活方式；强化服务措施，着力解决群众反映的就医流程“三长一短”问题；建立医疗纠纷第三方调解机制，维护医患双方合法权益。推进市级“三馆”合一工程和县级“两馆”达标建设，市传媒大厦投入使用；在全市公共场所和大型商业区开通Wi-Fi；启动县级数字影院和“百村影院”工程，完成107个中心村的文化广场、文化活动室建设任务。各级体育场馆和有条件的校园健身场地定期向社会免费开放，逐步形成城乡“十五分钟健身圈”。加强食品药品市场监管，争创国家级食品安全城市。提升流动人口基本公共服务均等化水平。强化社会治理。深入实施社区“双百”示范工程，改善40个城区社区用房条件。提高防范预警和区域应急联动能力，坚决遏制重特大安全生产事故发生。完善社会治安立体防控体系，依法严厉打击各类违法犯罪活动。整合便民热线，建成综合服务新平台。培育和践行社会主义核心价值观，持续提升城市文明程度。加强社会性普法体系建设，拓宽法律援助渠道，推进基本公共法律服务均等化、便民化。实现人民调解、行政调解、司法调解联动，及时就地解决群众的合理诉求。深入开展“双拥在基层”活动，争创全国双拥模范城“八连冠”。

　　三、推进依法行政

　　加快建设职能科学、权责法定、执法严明、公开公正、廉洁高效的法治政府，努力营造依法行政、依法办事的法治环境。

　　(一)依法履行政府职能。增强法治意识，坚持法无授权不可为、法定职责必须为，自觉运用法治思维、法治方式和法治手段，行使权力、履行职责。牢记法律红线不可逾越、法律底线不可触碰，带头遵守法律、执行法律，引领和带动全社会形成办事依法、遇事找法、解决问题用法、化解矛盾靠法的良好法治环境。加强和改进行政复议、行政应诉工作。

　　(二)严格公正文明执法。增强权责对等意识，规范行政执法程序，细化量化行政裁量标准，防止和减少行政权力的缺失或滥用，实现行政执法过程中的程序合法、实质正义。加快执法重点向基层转移，推进行政统一执法。加大食品药品、安全生产、环境保护、征地拆迁等关系群众切身利益的重点领域执法和责任追究力度，维护群众合法权益。

　　(三)健全依法决策机制。增强制度规则意识，把公众参与、专家论证、风险评估、合法性审查、集体讨论决定作为重大行政决策的法定程序，落实重大决策终身责任追究制度。组建专家咨询智库机构，服务重大决策。完善政府涉法事项风险防范机制，普遍建立政府法律顾问制度，实行重大行政决策法制审核和风险评估。

　　(四)强化权力监督制约。增强自觉接受监督意识，主动接受人大法律监督、政协民主监督和监察审计行政监督。继续深化政务公开，充分利用微博、微信等新媒体，及时传递政务信息，畅通社会舆论监督渠道，回应社会关切，让“民评官”、“找麻烦”成为新常态。

　　(五)加强政府管理创新。不以旧思维解决新问题，不以老办法应对新常态，提高用改革思路和市场力量促进发展的能力和水平。始终把干事创业谋发展作为最大的责任，增强诚信意识，强化政府工作的系统性、专业性，做到恪守规矩、“清风一路”。不讲过头话、不办过头事，在为民服务、招商引资中体现政府公信力。从严教育、管理干部，认真解决庸政、懒政、怠政现象，在狠抓落实、推进发展中彰显政府执行力。

　　(六)加大反腐倡廉力度。坚持廉洁从政、从严治政，深入推进政府系统反腐倡廉惩防体系制度性建设，完善重点领域的业务规则设计，普遍开展制度廉洁性评估，依法设定权力、规范权力、制约权力、监督权力，从源头上预防和治理腐败。认真落实“一岗双责”，层层传递压力，层层明确责任，种好党风廉政建设和反腐败斗争的“责任田”，严肃查办各类违法违纪案件，始终保持惩治腐败的高压态势。

　　各位代表，新常态赋予新使命，新常态要有新作为。让我们在省委、省政府和市委的正确领导下，勇于创新、勇于担当，不断凝聚改革创新的正能量，不断激发促进发展的新活力，真抓实干，攻坚克难，共同铸就全市人民更加美好的梦想！
